# False-positive results of galactomannan assays in patients administered glucose-containing solutions

**DOI:** 10.1038/s41598-024-53116-x

**Published:** 2024-01-31

**Authors:** Dong Woo Shin, Jungwon Cho, Kyung Suk Choi, Jungwha Lee, Yunsang Choi, Seong Jin Choi, Sang-A Kim, Song Mi Moon, Eu Suk Kim, Hong Bin Kim, Kyoung Un Park, Yun Ji Hong, Kyoung-Ho Song

**Affiliations:** 1 Department of Laboratory Medicine, Seoul National University Bundang Hospital, Seongnam, Republic of Korea; 2 Department of Pharmacy, Seoul National University Bundang Hospital, Seongnam, Republic of Korea; 3 Department of Internal Medicine, Seoul National University Bundang Hospital, Seoul National University College of Medicine, Seongnam, Republic of Korea; 4 Department of Laboratory Medicine, Seoul National University College of Medicine, Seoul, Republic of Korea

**Keywords:** Microbiology, Health care, Medical research

## Abstract

Galactomannan (GM) is a polysaccharide cell wall component released by *Aspergillus* spp., and an immunoenzymatic GM assay is used for the diagnosis of invasive pulmonary aspergillosis. We evaluated the cause of strong positivity for GM in patients with no typical signs of aspergillosis. Repeat assays were performed using different instruments and reagent lots, but there were no differences in results among the assays. Patients with strongly positive GM results were investigated. Medication histories revealed that 14 of 23 patients had been administered total parenteral nutrition solution from one manufacturer and 4 patients had been administered dextrose solution from a different manufacturer before being tested. The results of GM assays conducted on samples of dextrose solution and the glucose fraction of the total parenteral nutrition solution were strongly positive, confirming the causes of the false-positive reactions. We hypothesize that a trace amount of GM was introduced into the glucose-containing solutions because glucoamylase, which is necessary for the saccharification step of glucose synthesis, was derived from *Aspergillus niger*. To enhance patient care and prevent unnecessary antifungal prescriptions, healthcare providers and manufacturers of healthcare products need to be aware of the possibility of false-positive reactions for GM.

## Introduction

Galactomannan (GM) is a polysaccharide cell wall component released by *Aspergillus* spp. during growth^1^. An immunoenzymatic sandwich microplate assay using a rat anti-galactofuranose monoclonal antibody (EB-A2), which is directed against GM, was approved by the United States Food and Drug Administration in 2003^2,3^. The GM assay has high sensitivity and moderate accuracy for the diagnosis of invasive aspergillosis in immunocompromised patients^1^.

Although the GM assay is used worldwide because it is easily performed and has good performance, false-positive results in patients with no clinical signs have been reported, including in those receiving piperacillin/tazobactam^4^, beta-lactams^5^, and other antibiotics^6^. Patients receiving total parenteral nutrition (TPN)^7^ or crystalloid solutions^8^ also had false-positive GM results. Moreover, patients with mucositis or an altered intestinal barrier showed false-positive GM results because of dietary factors^9,10^.

The number of patients positive by GM assay with a high index but without typical signs of pulmonary invasive aspergillosis suddenly increased in our hospital. We evaluated the cause of the false-positive GM results in these patients.

## Results

### Timeline

The timeline of this study is tabulated (Table 1). The laboratory was notified by medical departments of several patients suspected to have false-positive GM results. The assays were repeated using different instruments and reagent lots. The medication histories of patients with strongly positive GM results indicated that they had received TPN from manufacturer A before being tested. GM assays were performed on several lots of TPN solutions from manufacturers A (TPN-A) and B (TPN-B), and the glucose fraction of the TPN-A solution showed a strongly positive result. In addition, a patient with a false-positive result who had not been administered TPN-A before testing had been administered dextrose from manufacturer X (dextrose-X). A GM assay on dextrose-X showed a strongly positive result.

**Table 1:** Timeline of investigation of the cause of false-positive results for galactomannan assay.

Date	Action	Note
24 Nov	Raising an issue	Recent increase of patients with positive GM assay and high index but without typical signs of pulmonary invasive aspergillosis - Six patients from IMI and one patient from IMH Request to the laboratory for a check
25Nov	Repeat assays with different instrument	
29 Nov	Request for recent cases with positive GM assay	
30 Nov	Repeat assays with a reagent with another lot	The results did not differ according to reagent lot or instrument (Supplementary Table S1) Suspicious for drug interferences such as antibiotics → Investigation of medication records
1–5 Dec	Selection of recent cases with strongly positive GM assay (index > 5)	A total of 23 cases (from 11 Nov to 5 Dec) → Investigation of medication records from 1 Oct
1–6 Dec	Investigation of medication records of selected cases	TPN-A administration records in 15 cases (Fig. 1) → Analysis of TPN-A Literature review Contact with manufacturer A
6 Dec	Contact with manufacturer A	
7 Dec	Notification of prescription stop and alternative nutritional solutions in hospital	
7–8 Dec	GM assay by samples from TPN-A and several medical solutions	Strongly positive in glucose fraction of TPN-A (Table 2)
8 Dec	Conference (medical/pharmaceutical department & manufacturer) Notice in the hospital: false positive of GM assay by TPN-A Addition of comments when reporting results Manufacturer's notice for all hospitals supplied with TPN-A Recall processing and distribution of new products	The TPN-A-manufacturer recently changed the source of glucose to another company A trace amount of GM was contaminated during manufacturing a fermentation enzyme
12 Dec	Investigation of four patients with positive results without TPN-A administration before testing	5% dextrose from manufacturer X was commonly administered to these patients
13 Dec	GM assay by dextrose solution	Strongly positive, contact with the manufacturer X

*GM* galactomannan, *IMI* Division of Infectious Diseases, Department of Internal Medicine, *IMH* Division of Hematology, Department of Internal Medicine, *TPN-A* total parenteral nutrition solution from manufacturer A.

### Results of GM assays using different lots and instruments

GM assays were performed on two platforms using reagents of old and new lots (Supplementary Table S1). Although the sample index values were not identical, the results (positive or negative) of 16 samples did not differ according to reagent lot or instrument.

### Medication histories of patients with strongly positive results of GM assays

**Figure 1:**
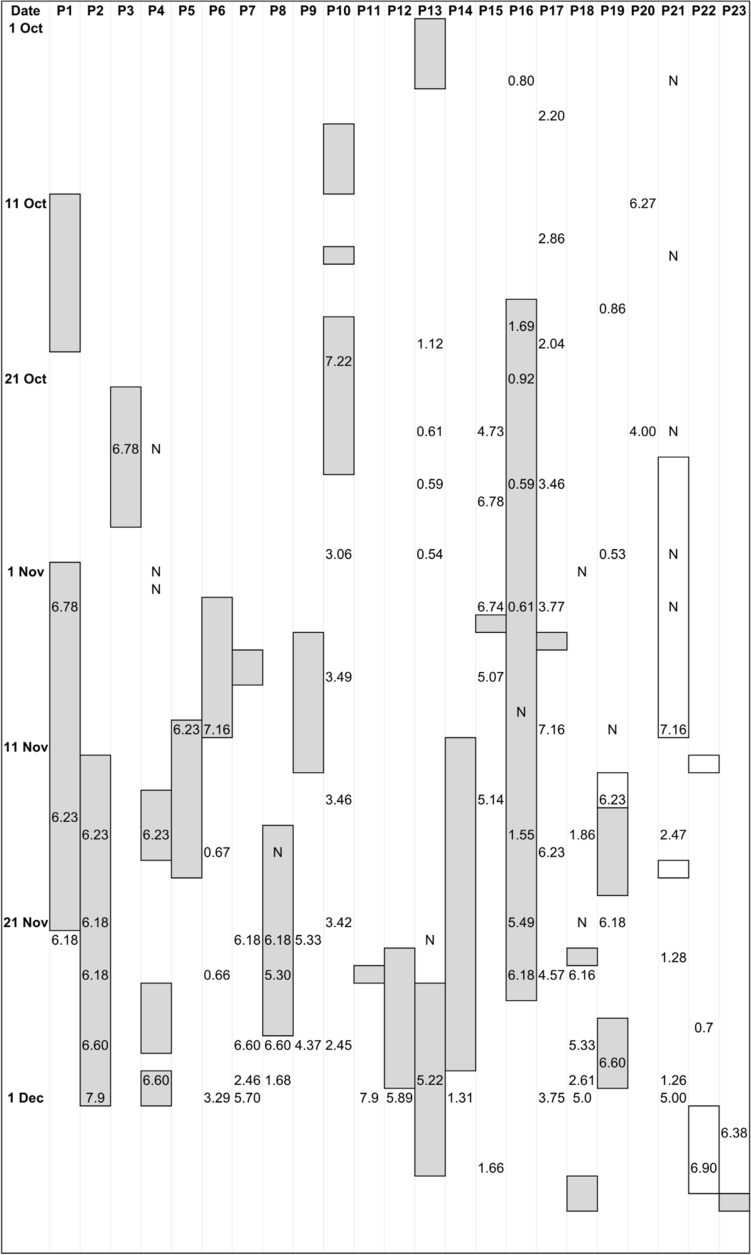
Galactomannan (GM) assay results and history of administration of total parenteral nutrition from manufacturer A (TPN-A) and 5% dextrose solution from manufacturer X (dextrose-X). Gray boxes, administration of TPN-A; white boxes, administration of 5% dextrose-X. Numbers indicate GM assay index values (N, negative). P1-P14, patients with false-positive results for GM due to administration of TPN-A prior to testing. P15-P18, patients with probable invasive pulmonary aspergillosis and an abrupt increase of index values after administration of TPN-A. P19-P22, false-positive results for GM due to administration of 5% dextrose-X prior to testing. P20 received 5% dextrose-X 16–18 September (not shown). P23, medication history in another hospital (22 October to 25 November) prior to transfer to our hospital was not available.

There were 23 patients with strongly positive (index > 5.0) results and/or suspicion of a false-positive result. Of these 23 patients, 20 received TPN-A, and it was administered prior to testing in 14 patients (Fig. 1). The diagnoses of these 14 patients were as follows: kidney transplant status (P1), hematologic malignancy patients who received bone marrow transplant (P2, P4, P7, P8, P9, P13, P14), lung cancer (P3, P12), interstitial lung disease (P5, P6), and pleural effusion (P10, P11). Despite the high GM index values, symptoms and chest computed tomography (CT) findings in those were not suggestive of invasive pulmonary aspergillosis (IPA). Also, 12 of the 23 patients received piperacillin/tazobactam but no correlation was found between the timing of administration and testing. Therefore, TPN-A was suspected to be the cause of the false-positive results.

### Results of GM assays on medical solutions

GM assays were performed on samples from TPN-A, TPN-B, dextrose-X, and dextrose from manufacturer Y (dextrose-Y) (Table 2). The results were positive for the glucose fractions of TPN-A in five of seven lots but negative for the other fractions of TPN-A and for the glucose fraction of TPN-B. TPN-A for a peripheral line showed higher index values than TPN-A for a central line. The GM assay result was positive for 5% dextrose-X but negative for 50% dextrose-Y.

**Table 2:** Results of galactomannan assays performed on nutrition fluids and their fractions.

Fluid*	Fraction	Index**	Result
TPN-A (C, lot 1)	Glucose	2.15, 2.35	Positive
	Amino acids & electrolytes	0.14, 0.15	Negative
	Lipids	0.23, 0.21	Negative
	Lipids (diluted)	0.30, 0.37	Negative
TPN-A (P, lot 1)	Glucose	8.45, 8.45	Positive
	Amino acids & electrolytes	0.24, 0.29	Negative
	Lipids	0.19, 0.22	Negative
	Lipids (diluted)	0.21, 0.20	Negative
TPN-B (lot 1)	Glucose	0.22, 0.19	Negative
TPN-A (C, lot 2)	Glucose	0.14, 0.15	Negative
TPN-A (C, lot 3)	Glucose	3.65, 3.75	Positive
TPN-A (C, lot 4)	Glucose	0.22, 0.19	Negative
TPN-A (P, lot 2)	Glucose	6.9, 6.9	Positive
TPN-A (P, lot 3)	Glucose	6.9, 6.9	Positive
5% Dextrose-X (lot 1)	–	8.52, 8.52	Positive
50% Dextrose-Y (lot 1)	–	0.16, 0.16	Negative

*TPN* total parenteral nutrition, *C* for central line, *P* for peripheral line.

*A, B, X, and Y are tentative names of manufacturers.

**Tests were performed in duplicate.

### GM results after discontinuing administration of TPN-A

**Figure 2:**
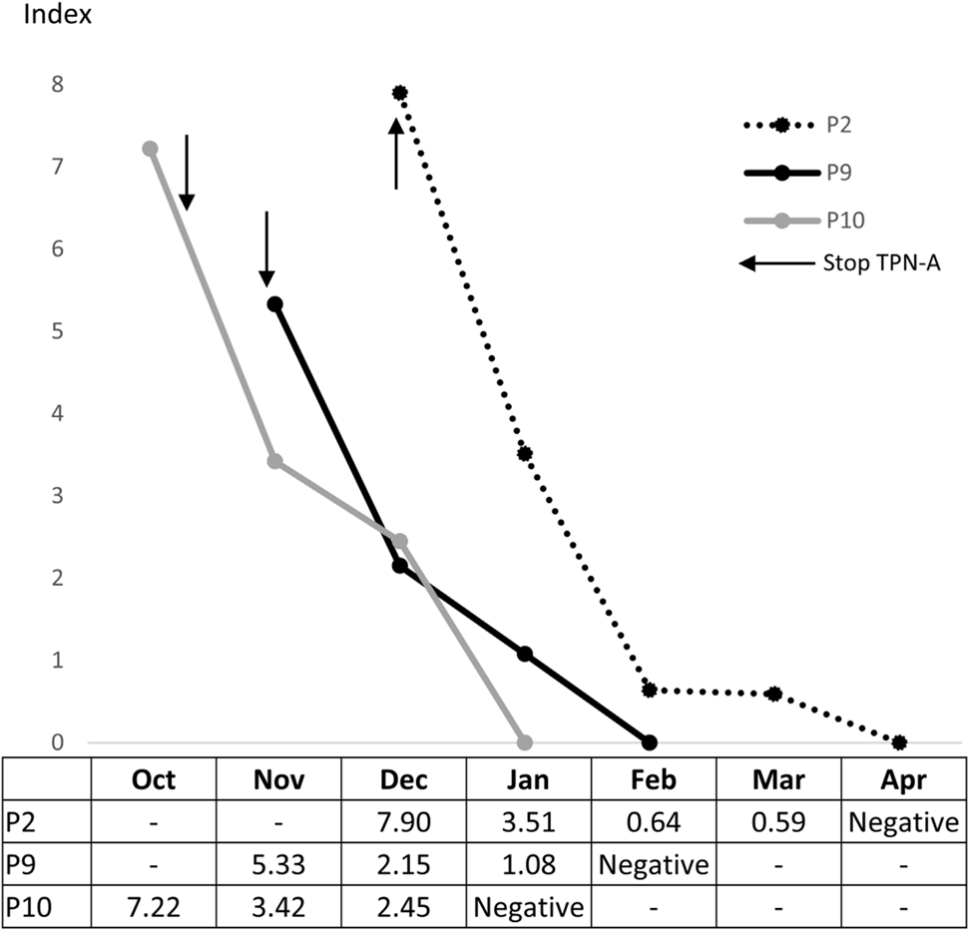
Cases in which galactomannan assay results were confirmed to turn negative after discontinuing administration of total parenteral nutrition from manufacturer A (TPN-A). P2, P9, and P10 were administered TPN-A for more than a week and discontinued on 1 December, 12 November, and 26 October, respectively. It took 125, 68, and 79 days, respectively, for the galactomannan assay results of P2, P9, and P10 to change to negative after cessation of TPN-A.

Of the 13 patients with strongly false-positive results for GM due to administration of TPN-A (P1-P13 in Fig. 1), 3 had GM results that turn negative. P1, P9, and P10 all were administered TPN-A for more than a week, and their GM results changed to negative 125, 68, and 79 days after discontinuation of administration, respectively (Fig. 2). The remaining 11 had no follow-up GM results until they became negative. GM results were followed up to 4 months after stopping prescription of TPN-A and 5% dextrose-X, and no false-positive cases occurred in our hospital.

## Discussion

*Aspergillus* spp. are common in the environment, and can cause IPA in immunocompromised patients such as recipients of hematopoietic stem cell transplants or solid organ transplants, patients undergoing chemotherapy, and those with human immunodeficiency virus infection^11^. Cases with positive GM results in this study were suspected to be false positives because the clinical manifestations of most of the patients did not indicate IPA. The medication histories of the patients revealed that they had been administered glucose-containing solutions, TPN-A and dextrose-X, which were identified as the causes of the false-positive results, proven by GM assays using direct samples of these solutions.

Among the 23 patients, 13 were strongly false-positive for GM after being administered TPN-A (P1-13 in Fig. 1). Follow-up GM assays were considered unnecessary in most of them, and they were not prescribed sufficiently until the results became negative. In 3 patients followed until GM results changed to negative, time to negativity after discontinuation of TPN-A administration ranged 68–125 days (Fig. 2). However, a previous study reported that the average time to negativity was estimated to be 5.5 days and the half-life of GM was estimated to be 2.4 days^5^. This difference is likely because the patients were administered TPN-A for a prolonged period and their index values of GM assays were high (> 5.0), and the metabolic pathways and structures of GM originating from infectious *Aspergillus* spp. and glucose-manufacturing may be different. P13 and P16 were weakly positive for GM (index < 2.0) in October due to administration of TPN-A, but strongly positive in November. This was because manufacturer A changed the enzyme used to manufacture the glucose-containing solution in November. For the same reason, the index values of TPN-A differed among lots (Table 2). The index value of P14 was low, because this patient received TPN-A for a central line, which had a lower index value than TPN-A for a peripheral line. Patients who had received 5% dextrose-X (P19-22) were strongly positive for GM and remained positive for more than 1 week after cessation of 5% dextrose-X administration. P23 had a history of inpatient treatment at another hospital before being transferred to our hospital and may have received TPN-A or dextrose-X at that hospital.

The glucose in medical solutions is produced from starch in a multi-step process. The saccharification step uses glucoamylase, an enzyme from *A*. *niger* or *Bacillus licheniformis*. Glucoamylase from *A. niger* is used in the industrial production of glucose from raw starch or soluble oligosaccharides because it is thermostable and highly active in near-neutral pH environments^12^. If there is a problem extracting or purifying glucoamylase from *A. niger*, GM from the cell wall of *A. niger* may be introduced, potentially explaining GM positivity in glucose-containing solutions. Manufacturers A and X recently changed their glucoamylase suppliers, resulting in false-positive GM results in subsequent lots of TPN-A and dextrose-X. However, the glucoamylase supplier reported that the enzyme was not contaminated with viable fungi. Manufacturer A notified customers in South Korea of this problem and recalled the problematic lots of TPN-A. The possibility of false-positive results for GM by TPN-A was relayed to the hospital staff, TPN-A prescription was suspended, and comments were added to the results of GM assays.

Several causes of false-positive GM results were reported previously. Not only glucoamylase, but also citric acids are prepared from *A. niger*. A variety of anticoagulants and additive solutions used in platelet units contain citric acids, and there was a false-positive results of GM after platelet transfusion^13^. GM from *Penicillium* spp. is used in the manufacture of antibiotics, such as piperacillin/tazobactam and amoxicillin/clavulanate, and has been reported as a cause of false-positive results^14^. Other causes of false-positive GM results include crystalloid solutions, antibiotics, and dietary factors^4–10^. False-positive GM results may lead to antifungal prescriptions and additional workup such as chest CT, resulting in unnecessary medical costs and even harm to patients. Even worse is that false-positive GM results in symptomatic patients with pulmonary lesions in CT may mask more serious diseases which have symptomatic overlap with IPA, such as mucormycosis. To enhance patient care, healthcare providers and manufacturers of healthcare products need to be aware of the possibility of false-positive results for GM.

This study had several limitations. Other diagnostic tests for IPA, such as beta-D-glucan, *Aspergillus* lateral-flow assay, culture, and polymerase chain reaction^15^, were not performed. True or false positivity for *Aspergillus* spp. can be confirmed using other diagnostic tools. If the GM assay is problematic or not available, other diagnostic methods should be considered. Also, in most patients, GM assay results were not followed until they turned negative. As an observational study, this study was based on the dates of prescription of the medical solution and of the GM assay, and no patient intervention was performed. A systematic trial involving a regular follow-up after administration of several glucose-containing solutions is thus needed but may be controversial because of the benefit-risk balance.

In conclusion, patients strongly positive for GM without typical signs of IPA had been administered glucose-containing solutions from several manufacturers. These solutions were confirmed to be the causes of the false-positive results. Healthcare providers and manufacturers of medical products should be aware of causes of false-positive results for GM. If a diagnostic test for *Aspergillus* is problematic, use of alternative diagnostic methods is recommended.

## Methods

### Repeat GM assays

Repeat GM assays were performed on samples from patients suspected by physicians to have false-positive results and on samples from patients with positive or borderline values from 21 to 24 November, 2022. GM assays (Platelia *Aspergillus* Ag, Bio-Rad, Hercules, CA, USA) were performed on the GEMINI (Diatron, Budapest, Hungary) and Multiskan FC Microplate Photometer (Thermo Fisher Scientific, Waltham, MA, USA) platforms. To confirm reagent denaturation or contamination, GM assays were performed on old and new lots. An index value of ≥ 0.5 was classified as positive for GM.

### Medication records

Patients who had strongly positive (index > 5.0) results or suspicion by physicians of false positivity for GM assays from 11 November to 5 December 2022 were included in the analysis. Medical histories and medication records from 1 October to 8 December were investigated to identify commonly prescribed medications.

### GM assays on samples from nutritional solutions

Investigation of medication records revealed that the patients, who were strongly positive for GM, had received TPN-A. GM assays were performed on samples of the TPN-A and TPN-B, and dextrose-X and dextrose-Y. Because TPN solutions were divided into glucose, amino acid and electrolytes, and lipids fractions, GM assays were performed on samples of each.

### Follow-up GM results after discontinuing administration of TPN-A

To confirm whether the GM results changed to negative after discontinuation of TPN-A administration, the GM results of patients suspected to have false-positive results were tracked. The time taken for GM results to become negative after TPN-A discontinuation was investigated.

### Ethical approval

This study was approved by the Institutional Review Board of Seoul National University Bundang Hospital. The need for informed consent was waived by the Institutional Review Board of Seoul National University Bundang Hospital (study number: B-2301-807-101). All methods were performed in accordance with the Declaration of Helsinki.

### Supplementary Information

Supplementary Information.

## Data Availability

The data supporting this study are included in this published article and its Supplementary Information.

## References

[1] Pfeiffer CD, Fine JP, Safdar N (2006). Diagnosis of invasive aspergillosis using a galactomannan assay: A meta-analysis. Clin. Infect. Dis..

[2] Bio-Rad. Platelia *Aspergillus* Ag insert. Preprint at https://commerce.bio-rad.com/webroot/web/pdf/inserts/CDG/en/62794_881115_EN.pdf (2013).

[3] Berkeley, L., Dixon, C. & Bala, S. Detection of Galactomannan in Serum by Platelia^TM^*Aspergillus* Enzyme-linked Immunosorbent Assay (BioRad Laboratories and Sanofi Diagnostics). Preprint at https://www.fda.gov/media/90001/download (2011).

[4] Demiraslan H (2017). Assessing the risk of false positive serum galactomannan among patients receiving piperacillin/tazobactam for febrile neutropenia. Med. Mycol..

[5] Aubry A (2006). Occurrence and kinetics of false-positive *Aspergillus* galactomannan test results following treatment with beta-lactam antibiotics in patients with hematological disorders. J. Clin. Microbiol..

[6] Boonsarngsuk V, Niyompattama A, Teosirimongkol C, Sriwanichrak K (2010). False-positive serum and bronchoalveolar lavage *Aspergillus* galactomannan assays caused by different antibiotics. Scand. J. Infect. Dis..

[7] Blijlevens NM, Donnelly JP, Meis JF, Verweij PE, de Pauw BE (2002). *Aspergillus* galactomannan antigen levels in allogeneic haematopoietic stem cell transplant recipients given total parenteral nutrition. Transpl. Infect. Dis..

[8] Hage CA, Reynolds JM, Durkin M, Wheat LJ, Knox KS (2007). Plasmalyte as a cause of false-positive results for *Aspergillus* galactomannan in bronchoalveolar lavage fluid. J. Clin. Microbiol..

[9] Ansorg R, van den Boom R, Rath PM (1997). Detection of *Aspergillus* galactomannan antigen in foods and antibiotics. Mycoses.

[10] Gangneux JP, Lavarde D, Bretagne S, Guiguen C, Gandemer V (2002). Transient aspergillus antigenaemia: Think of milk. Lancet.

[11] Weber DJ, Peppercorn A, Miller MB, Sickbert-Benett E, Rutala WA (2009). Preventing healthcare-associated *Aspergillus* infections: Review of recent CDC/HICPAC recommendations. Med. Mycol..

[12] Lee J, Paetzel M (2011). Structure of the catalytic domain of glucoamylase from *Aspergillus* niger. Acta Crystallogr. Sect. F Struct. Biol. Cryst. Commun..

[13] Martín-Rabadán P, Gijón P, Alonso Fernández R, Ballesteros M, Anguita J, Bouza E (2012). False-positive *Aspergillus* antigenemia due to blood product conditioning fluids. Clin. Infect. Dis..

[14] Wheat LJ, Walsh TJ (2008). Diagnosis of invasive aspergillosis by galactomannan antigenemia detection using an enzyme immunoassay. Eur. J. Clin. Microbiol. Infect. Dis..

[15] Hoenigl M (2014). Performance of galactomannan, beta-d-glucan, *Aspergillus* lateral-flow device, conventional culture, and PCR tests with bronchoalveolar lavage fluid for diagnosis of invasive pulmonary aspergillosis. J. Clin. Microbiol..

