# Supplementary material for: False-positive results of galactomannan assays in patients administered glucose-containing solutions
Source: Sci Rep. 2024 Jan 31;14:2552. doi: 10.1038/s41598-024-53116-x (PMC10827775; doi:10.1038/s41598-024-53116-x)
Supplement: Supplementary file 1 — Supplementary Information. [file 41598_2024_53116_MOESM1_ESM.docx]

Supplementary Table S1. Results of galactomannan assays performed on two platforms using old and new reagent lots.

| **Instrument** | **GEMINI** | **GEMINI** | **Multiskan** | **GEMINI** | **Result** |
| --- | --- | --- | --- | --- | --- |
| **Reagent lot** | **Old** | **Old (re)** | **Old** | **New** |  |
| **Case No.** | **Sample Index** | | | |  |
| S1 | 0.46 | 0.52 | 0.53 | NT | Positive |
| S2 | 6.18 | 8.63 | 10.63 | 6.23 | Positive |
| S3 | 5.49 | 6.09 | 6.04 | 4.61 | Positive |
| S4 | 6.18 | 8.63 | 11.75 | 6.23 | Positive |
| S5 | 6.18 | 8.63 | 10.56 | 6.23 | Positive |
| S6 | 5.33 | 7.17 | 7.13 | 5.46 | Positive |
| S7 | 6.18 | 8.63 | 10.14 | 6.23 | Positive |
| S8 | 6.18 | 8.63 | 11.07 | 6.23 | Positive |
| S9 | 0.49 | 0.54 | 0.59 | NT | Positive |
| S10 | 6.18 | 8.63 | 9.87 | 6.23 | Positive |
| S11 | 5.30 | 6.76 | 6.93 | 5.59 | Positive |
| S12 | 6.16 | 8.63 | 9.31 | 6.03 | Positive |
| S13 | 6.18 | 8.63 | 10.51 | 6.23 | Positive |
| S14 | 6.18 | 6.64 | 6.48 | 6.23 | Positive |
| S15 | 0.43 | 0.39 | 0.36 | 0.45 | Negative |
| S16 | 1.94 | 1.67 | 1.63 | 2.04 | Positive |

NT, not tested
